# Paradoxical Immune Responses in Non-HIV Cryptococcal Meningitis

**DOI:** 10.1371/journal.ppat.1004884

**Published:** 2015-05-28

**Authors:** Anil A. Panackal, Simone C. Wuest, Yen-Chih Lin, Tianxia Wu, Nannan Zhang, Peter Kosa, Mika Komori, Andrew Blake, Sarah K. Browne, Lindsey B. Rosen, Ferry Hagen, Jacques Meis, Stuart M. Levitz, Martha Quezado, Dima Hammoud, John E. Bennett, Bibi Bielekova, Peter R. Williamson

**Affiliations:** 1 Laboratory of Clinical Infectious Diseases, NIAID, NIH, Bethesda, Maryland, United States of America; 2 Division of Infectious Diseases, Department of Medicine, F. Hebert School of Medicine, Uniformed Services University of the Health Sciences (USUHS), Bethesda, Maryland, United States of America; 3 Neuroimmunological Diseases Unit, Neuroimmunology Branch, National Institute of Neurological Diseases and Stroke (NINDS), National Institutes of Health (NIH), Bethesda, Maryland, United States of America; 4 Department of Medical Microbiology and Infectious Diseases, Canisius-Wilhelmina Hospital, Nijmegen, The Netherlands; 5 Department of Medical Microbiology, Radboudumc, Nijmegen, The Netherlands; 6 Department of Medicine, University of Massachusetts Medical School, Worcester, Massachusetts, United States of America; 7 Laboratory of Pathology, Center for Cancer Research, National Cancer Institute, National Institutes of Health, Bethesda, Maryland, United States of America; 8 Center for Infectious Disease Imaging, Radiology and Imaging Sciences, National Institutes of Health/Clinical Center, Bethesda, Maryland, United States of America; University of Birmingham, UNITED KINGDOM

## Abstract

The fungus *Cryptococcus* is a major cause of meningoencephalitis in HIV-infected as well as HIV-uninfected individuals with mortalities in developed countries of 20% and 30%, respectively. In HIV-related disease, defects in T-cell immunity are paramount, whereas there is little understanding of mechanisms of susceptibility in non-HIV related disease, especially that occurring in previously healthy adults. The present description is the first detailed immunological study of non-HIV-infected patients including those with severe central nervous system (s-CNS) disease to 1) identify mechanisms of susceptibility as well as 2) understand mechanisms underlying severe disease. Despite the expectation that, as in HIV, T-cell immunity would be deficient in such patients, cerebrospinal fluid (CSF) immunophenotyping, T-cell activation studies, soluble cytokine mapping and tissue cellular phenotyping demonstrated that patients with s-CNS disease had effective microbiological control, but displayed strong intrathecal expansion and activation of cells of both the innate and adaptive immunity including HLA-DR+ CD4+ and CD8+ cells and NK cells. These expanded CSF T cells were enriched for cryptococcal-antigen specific CD4+ cells and expressed high levels of IFN-γ as well as a lack of elevated CSF levels of typical T-cell specific Th2 cytokines -- IL-4 and IL-13. This inflammatory response was accompanied by elevated levels of CSF NFL, a marker of axonal damage, consistent with ongoing neurological damage. However, while tissue macrophage recruitment to the site of infection was intact, polarization studies of brain biopsy and autopsy specimens demonstrated an M2 macrophage polarization and poor phagocytosis of fungal cells. These studies thus expand the paradigm for cryptococcal disease susceptibility to include a prominent role for macrophage activation defects and suggest a spectrum of disease whereby severe neurological disease is characterized by immune-mediated host cell damage.

## Author Summary

*Cryptococcus* is an important cause of fungal meningitis with significant mortality globally. Susceptibility to the fungus in humans has been related to T-lymphocyte defects in HIV-infected individuals, but little is known about possible immune defects in non HIV-infected patients including previously healthy individuals. This latter group also has some of the worst response rates to therapy with almost a third dying in the United States, despite available therapy. Here we conducted the first detailed immunological analysis of non-HIV apparently immunocompetent individuals with active cryptococcal disease. In contrast to HIV-infected individuals, these studies identified a highly activated antigen-presenting dendritic cell population within CSF, accompanied by a highly active T-lymphocyte population with potentially damaging inflammatory cytokine responses. Furthermore, elevated levels of CSF neurofilament light chains (NFL), a marker of axonal damage in severe central nervous system infections suggest a dysfunctional role to this acute inflammatory state. Paradoxically, CSF macrophage proportions were reduced in patients with severe disease and biopsy and autopsy samples identified alternatively activated tissue macrophage populations that failed to appropriately phagocytose fungal cells. Our study thus provides new insights into the susceptibility to human cryptococcal disease and identifies a paradoxically active T-lymphocyte response that may be amenable to adjunctive immunomodulation to improve treatment outcomes in this high-mortality disease.

## Introduction

*Cryptococcus neoformans* is an important cause of fatal meningoencephalitis in both those immunosuppressed from transplant conditioning or HIV/AIDS, as well as in previously healthy individuals. While AIDS-related cases represent the bulk of disease burden worldwide [1] with mortality approaching 60% in the developing world [2,3] and 20% in the developed world [4], non-HIV related cryptococcosis is a significant source of mortality and morbidity in the developed world, accounting for approximately a third of cases [5], with up to 30% mortality despite optimal therapy [4,6]. These mortality figures are derived from unselected cohorts in routine clinical settings and not clinical trials. In HIV-related disease where fungal burdens are high and cellular immunity low, recent approaches have sought to improve microbiological clearance from the CSF, an important prognostic marker [7]. These strategies have combined fungicidal drugs [8] or adjunctive cytokines such as interferon-γ (IFN-γ) [9,10]. The latter approach seeks to boost Th1-polarizing immunity, an immunological marker of survival during initial therapy [11]. In non-HIV-related disease, CSF fungal loads and effective microbiological clearance have similarly been associated with favorable outcomes [12]. However, little data is available regarding the immune milieu of these patients that could guide treatment, especially in severe or refractory cases. This has led to varying approaches for severe disease, including the use of immune intensifying regimens such as adjunctive IFN-γ [13], or immune-suppressive therapies such as steroids in the case of infections with *C*. *gattii* in previously healthy individuals [14]. Indeed, having too much or too little of a host immune response to infection may cause significant pathology [15,16]. Steroids have also been used in HIV-related cryptococcal meningoencephalitis with or without immune reconstitution syndrome (cIRIS), an aggravated paradoxical immune response to the pathogen in the setting of microbiologic control after restoration of T-cell activity from anti-retroviral therapy [17–19]. cIRIS is characterized by increases in immunoregulatory natural killer cells and IFN-γ-dependent factors such as monocyte attractant CXC motif chemokine 10 [20]. A clinical syndrome similar to cIRIS has also been reported in transplant recipients after reductions in immunoconditioning [21]. cIRIS-like syndromes are particularly problematic in cryptococcal meningitis, as the closed compartment of the brain within the skull allows little expansion with inflammation or cerebral edema. However, it is unclear whether the immunological response in cIRIS-like syndromes is similar in other cryptococcal patients.

Thus, given the controversies in management of severe cases of cryptococcal meningitis and an unexpected response to steroids in 2 sentinel cases under our care, more detailed, host-specific immune characterizations were sought to suggest a basis for rational treatment design in this complex disease. To this end, we conducted the first detailed immunological analysis of soluble and cellular responses to *Cryptococcus* in both blood and CSF in a consecutive cohort of non-HIV, non-transplant individuals with no comorbidities or iatrogenic immunosuppression who developed severe central nervous system disease (s-CNS). Despite minimal doses of steroids for maintenance of mental status that may have declined due to cerebral edema, immunophenotyping of s-CNS patients during therapy demonstrated a CNS compartmentalized, marked increase in activated T cell and NK cell populations, accompanied by elevated soluble IFN-γ, interferon-generating cytokines IL-18, and interferon-induced chemokines CXCL10, as compared to a cohort having non-CNS disease (non-CNS) or healthy donors (HD). Ex vivo T-cell activation with cryptococcal antigen-loaded dendritic cells (DCs) also demonstrated a compartmentalized Th1 response with increased CD4+ and CD8+ IFN-γproduction, thus providing a mechanism for the clinical deterioration and response to steroids. However, the present studies also demonstrated reduced monocyte and myeloid dendritic cells in the CSF and an M2 bias in tissue-infiltrating macrophages on examination of biopsy and autopsy specimens from patients with s-CNS disease, suggesting downstream defects in myeloid activation. These data thus identify a paradoxical Th1-biased CSF immune response in severe CNS cryptococcal disease after microbiological control, suggesting further study to investigate the role for adjunctive T-cell immunosuppressive therapy in this high mortality disease.

## Results

### Steroid-responsiveness of s-CNS disease in two previously healthy individuals provides a rationale for immunophenotyping studies

While the severity of the clinical conditions precluded randomization to steroid treatment, clinical responses to steroids (prednisone 1 mg/kg/d) were observed in two patients who developed deteriorating mental status despite effective fungicidal therapy and negative CSF fungal cultures. This provided a rationale for detailed immune studies, subsequently performed on 17 consecutive patients with CNS disease (Table 1) defined by positive cryptococcal cultures or antigens at the time of diagnosis. The subgroup excluded patients with previous immunosuppression or known risk factors for cryptococcal disease such as idiopathic CD4 lymphopenia [22].

**Table 1:** Patient characteristics of 17 severe central nervous system cryptococcosis (s-CNS) cases and 6 non-CNS cases.*

*Variable*	s-CNS (n = 17)	non-CNS (n = 6)
Median Age (years)	54 [IQR:44.0–59.0]	47.5 [IQR:35.3–56.0]
Female	7	4
Race:		
White	12	5
Black	1	0
Hispanic	1	0
Asian	3	1
Geographic Region†:		
Northeast	2	2
Midwest	0	0
South	9	3
Pacific Northwest	2	0
West	4	1
Antecedent Comorbidities:		
Diabetes mellitus	3	1
Hepatitis	1	1
Atopic Dermatitis/Asthma/Sinusitis	4	1
Thyroid disorder	2	1
Obesity	2	2
Smoking	2	1
Seizure disorder	1	0
Mucosal infection	2	0
None	5	1
Cryptococcal Species:		
*C*. *gattii*	5	1
*C*. *neoformans*	10	4
Unconfirmed	2	1
Median CD4+ Tcell count, cells/μl	526[IQR:324.0–828.6]	1241.5[IQR:862.2–1568.2]
Median CD8+ Tcell count, cells/μl	296[IQR:141.0–367.1]	392.0[IQR:337.8–487.4]
Median CSF Leukocyte Count per mm3	34 [IQR:13.0–57.3]	2 [IQR: 0.5–2.0]
Median CSF Cryptococcal Antigen titer	1:64[0–1:1024]	negative
Median Serum Cryptococcal Antigen titer	1:64 [0–1:32768]	0 [0–1:64]
Median Serum C-reactive Protein (mg/L)	3.78[IQR:1.39–8.18]	3.15[IQR:1.68–5.01]
Median Time from Symptom Onset to Diagnosis (days)	61 [IQR:18.0–133]	92 [IQR:50–146.5]
Median Time from Diagnosis to Study (days)	145[IQR:113.0–400.0]	798.5[IQR:666.5–1336.2]
Median Prednisone equivalent dose at Study (mg)	2.5 [IQR:0–30]	0
Shunt	12	N/A
Median Time of Last Dose of Amphotericin B until Study (days)	30 [IQR: 5.5–337]	N/A
Antifungal at time of Study:		
Lipid Amphotericin B	2	0
Voriconazole	5	0
Fluconazole	10	3
None	0	3
Signs and Symptoms at Time of Study:		
Fever	1	2
Mental Status Changes	7	0
Malaise	5	0
Headache	5	1
Visual Deficits	6	0
Sensorineural hearing loss	4	0
Balance problems	5	0
Focal Neurologic Signs	3	0
Nausea/Vomiting	1	0
Chronic Cough	0	0
Chest/Back Pain	1	2
Dyspnea	1	1
Asymptomatic	0	1

* = values at time of analysis

^†^ = categories modified from Agency for Healthcare Research and Quality

N/A = not applicable

IQR = inter-quartile range

**Fig 1:**
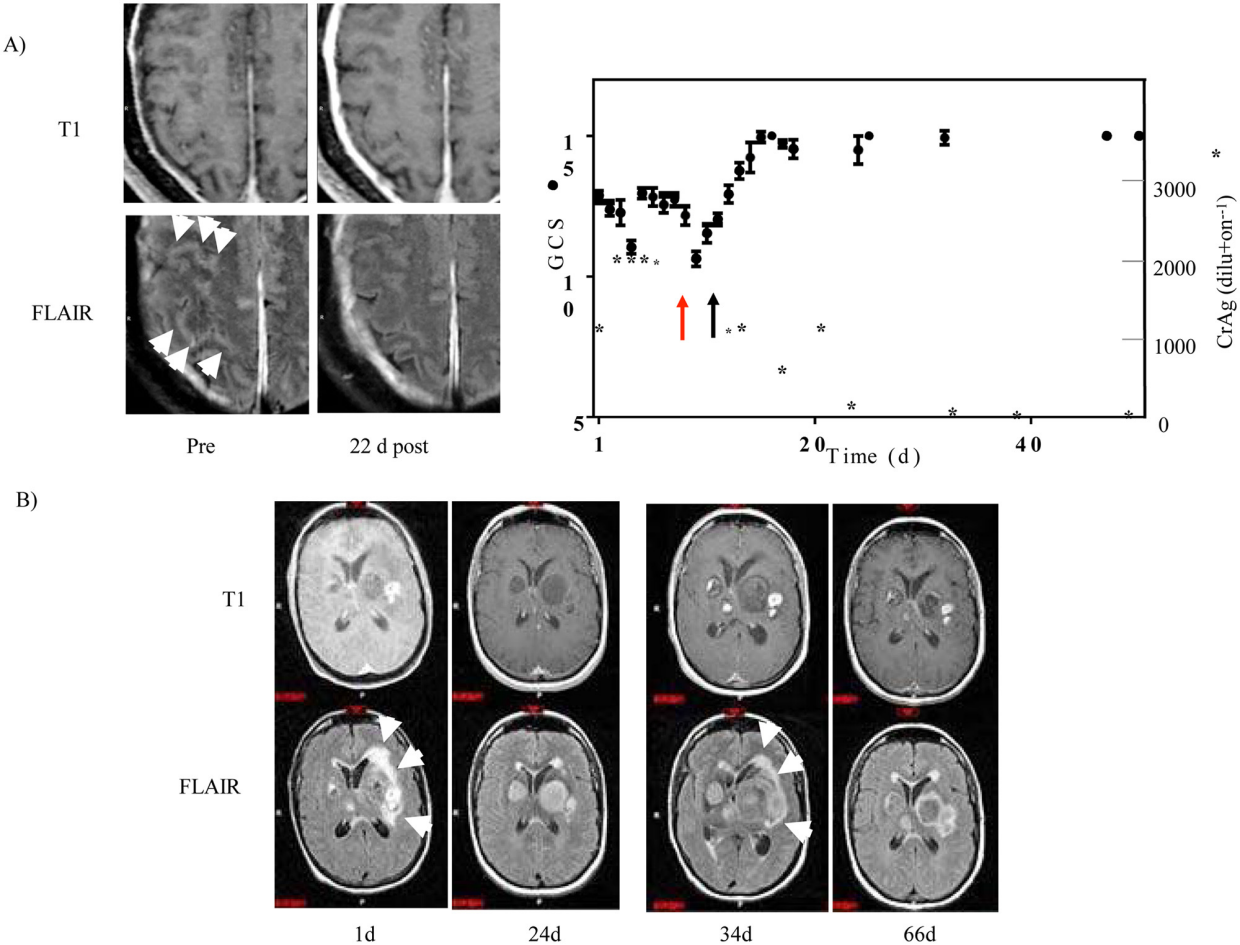
Steroid-responsiveness of s-CNS cryptococcosis in previously healthy patients. A) Patient 1: Left panel, Enhanced T1 (upper row) and FLAIR-weighted MRI images (lower row) pre-steroid (pre) and 22 days post (22 d post) steroid treatment. White arrowheads point to increased (bright) signal within the sulci on enhanced FLAIR imaging. Right panel, Ten day average Glasgow coma score (GCS) (•) +/- SEM (N > 20 for each value) and CSF cryptococcal latex antigen (*). Red arrow indicates time of initiation of treatment with IFN-γand black arrow indicates initiation of steroid therapy. B) Patient 2: Enhanced T1 (upper row) and FLAIR (lower row) MRI images are displayed, corresponding to the indicated day of admission (d). White arrows point to areas of edema surrounding the basal ganglia lesions. Days 1 and 34 were prior to steroid course #1 and #2, respectively. Day 24 and 66 were post steroid course #1 and #2, respectively.

The first steroid-responsive patient was a 58 year-old male who presented with dizziness, hearing loss and a left-sided facial droop. CSF cultures grew *C*. *neoformans*, identified on L-canavanine glycine bromothymol blue (CGB) media. The patient was treated with 6 weeks of intravenous amphotericin B lipid complex and flucytosine followed by fluconazole, but required placement of a ventriculoperitoneal shunt for deteriorating mental status and persistently elevated intracranial pressures. The patient was then transferred to the NIH clinical center, having a GCS of 13. Brain MRI scanning showed increased signal intensity (bright signal) on enhanced fluid attenuation inversion recovery (FLAIR) imaging within the sulci, especially the right central sulcus (Fig 1A). This patient was treated on day 1 with liposomal amphotericin B and flucytosine without improvement over a 2 month period with 10 day GCS mean scores ranging from 11–13, prompting a trial of recombinant interferon-γ 1b (red arrow in Fig 1A), but the patient developed hypersomnolence with significant worsening of mean GCS over the ensuing 10 days (pre: 12.2 +/-0.3 (SEM), N = 23; post: 10.6 +/- 0.3, N = 19; p = 0.001). As a result, recombinant interferon-γ 1b was discontinued and corticosteroids (prednisone 1 mg/kg/d) were added, after a 1 week ‘washout’ of IFN-γ, to the standing regimen. After this addition, the patient showed progressive improvement in mental status over the next 40 day period (10 day GCS, pre: 10.6 +/-0.3 (SEM), N = 19; 40 day post: 13.7 +/- 0.4, N = 21; p < 0.001) and normalization of GCS in 60 days (Fig 1B). Unfortunately, this patient developed residual bilateral cranial nerve VII and VIII palsies prior to steroid therapy, resulting in corneal scarring with consequential blindness and deafness, and gait disturbance and urinary retention from cryptococcal cauda equina syndrome.

The second patient was a 46 year-old female from Washington State who presented with intermittent fevers, worsening headaches, and altered mental status. MRI of the brain with gadolinium demonstrated multiple enhancing mass lesions in the left basal ganglia with the largest measuring approximately 3.5 x 2.8 centimeters. Adjacent smaller but more intensely enhancing lesions were seen in the left basal ganglia as well as throughout the brain, including multiple smaller lesions in the right posterior frontal lobe, left insula, right thalamus, left temporal lobe and left cerebellum (Fig 1B). The left basal ganglia lesions were associated with surrounding edema, best appreciated on FLAIR images as bright signal around the lesions, with secondary mass effect and slight impingement on the left lateral ventricle. Chest CT revealed an 8-cm right mid-lung mass, which was biopsied and grew *C*. *gattii*, genotype AFLP6A/VGIIa [23], consistent with the predominant outbreak strain found in the Pacific Northwest [24] and serum testing subsequently identified a functional anti-GMCSF autoantibody measured as previously described [25]. She was begun on liposomal amphotericin B, flucytosine and prednisone 50mg/d with improvement in mental status and MRI imaging at 24 days, which demonstrated decreased edema (decreased bright signal on FLAIR) in association with the left basal ganglia lesions and reduction in left lateral ventricular obstruction (Fig 1B, Day 24 and S1 Fig). Steroids were discontinued on day 24 to minimize possible immune suppression at the outside hospital and the patient’s status again deteriorated by day 34, prompting intensive care unit admission. Of note, the patient continued treatment with amphotericin B, remaining with negative CSF fungal cultures. Repeat MRI imaging showed recurrence of edema surrounding the left basal ganglia lesions with increased mass effect on the left lateral ventricle and slight midline shift (Fig 1B, Day 34). After consultation with the NIH, dexamethasone equivalent to prednisone 50mg/d was again added to the amphotericin B regimen with improvement in mental status and decreased enhancement and ventricular obstruction on brain MRI over the following month (Fig 1B, Day 66, S1 Fig). The patient was transferred to the NIH on Day 85 and an attempt was made to reduce the prednisone dose to 25 mg, but mild mental status deterioration and increased lesion size and edema on brain MRI (S1 Fig, Day 99) prompted an increase in steroid dose after her lumbar puncture and immunophenotyping with mental status improvement to a GCS back to normal (10 day GCS, pre: 14.3 +/-0.2 (SEM), N = 43; 10 day post: 15.0 +/- 0.0, N = 28; p < 0.01). Subsequently, amphotericin B was replaced by fluconazole and steroids were slowly tapered over the following 8 months as the cryptococcal antigen declined and was accompanied by normalization of activities. Fortunately, this patient made marked recovery with ability to resume her activities of daily living but with some residual memory deficits.

### Study subjects

Subjects were participants in an observational cohort examining the host genetics and immunology of cryptococcal disease in previously healthy, non-HIV infected adults. Written informed consent was obtained and approved by the research ethics committee of the NIAID institutional review board. Seventeen consecutively recruited patients with culture or biopsy-proven CNS cryptococcal disease were studied. Severe disease was present in all 17 and was defined as patients demonstrating significant deteriorations in mental status (Glasgow Coma Score, GCS < 15) despite standard fungicidal therapies with amphotericin B-based regimens and 12 of 17 required ventriculoperitoneal shunting for persistently elevated intracranial pressures. The s-CNS patients had demonstrated initial responses to antifungal therapy as evidenced by improvement in clinical, CSF, and radiologic parameters but subsequently worsened along these parameters in the absence of microbiologic growth or another infectious process after induction antifungals. As shown in Table 1, the 17 patients with s-CNS had median age of 54 years, with normal CD4+ (359-1565/μl) and CD8+ (178-853/μl) T lymphocyte counts. Patients were studied while undergoing active treatment with anti-fungal regimens and had negative CSF cultures at the time of analysis and remained negative throughout treatment. Fourteen required adjunctive prednisone (1 mg/kg/d or less) for control of cerebral edema, which was either begun after immunophenotyping or was minimized prior to study as symptoms allowed with a median dose of 2.5 mg/d. Of note, all patients were screened for GMCSF autoantibodies, but only two were positive (both s-CNS with *C*. *gattii*). Comparator groups consisted of HD as well as a non-CNS infected cryptococcal cohort that submitted to a lumbar puncture for research purposes.

### s-CNS demonstrate intrathecal enrichment of innate and adaptive immune cells with elevated levels of CSF NFL

**Fig 2:**
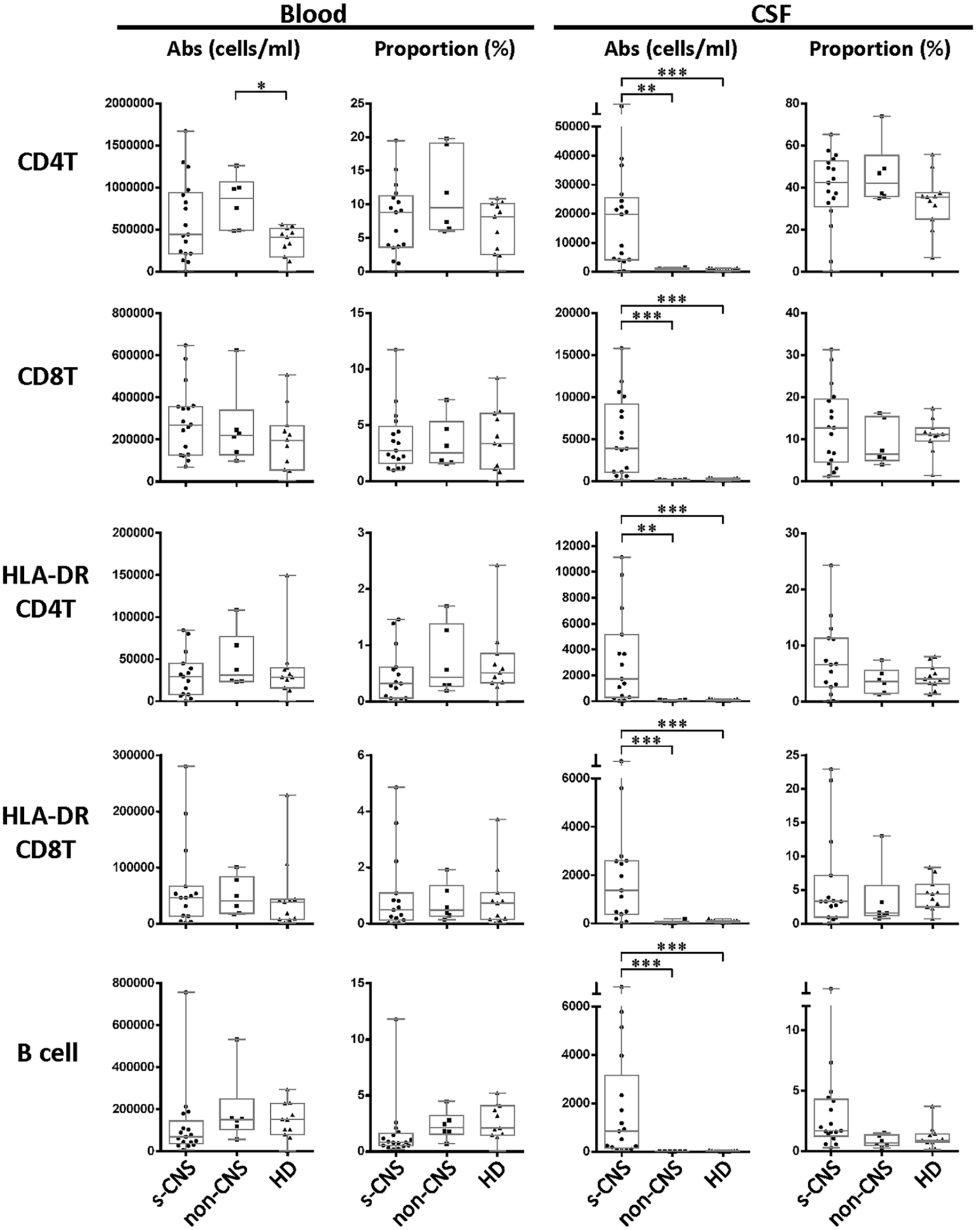
Immunophenotyping demonstrates increased absolute counts of CSF circulating activated HLA-DR+ CD4+ and CD8+ as well as—B-cells in s-CNS patients. Absolute numbers and proportions of activated CD4+ and CD8+ T lymphocytes (HLA-DR+),—and B lymphocytes were assessed in fresh CSF or blood by flow cytometry as described in Materials and Methods in patients with s-CNS disease (N = 17), non-CNS disease (N = 6), and healthy donors (HD; N = 11). Proportions are calculated from the total CD45+ cells. *0.01≤p<0.05; **0.001≤p<0.01; ***0.0001≤p<0.001.

**Fig 3:**
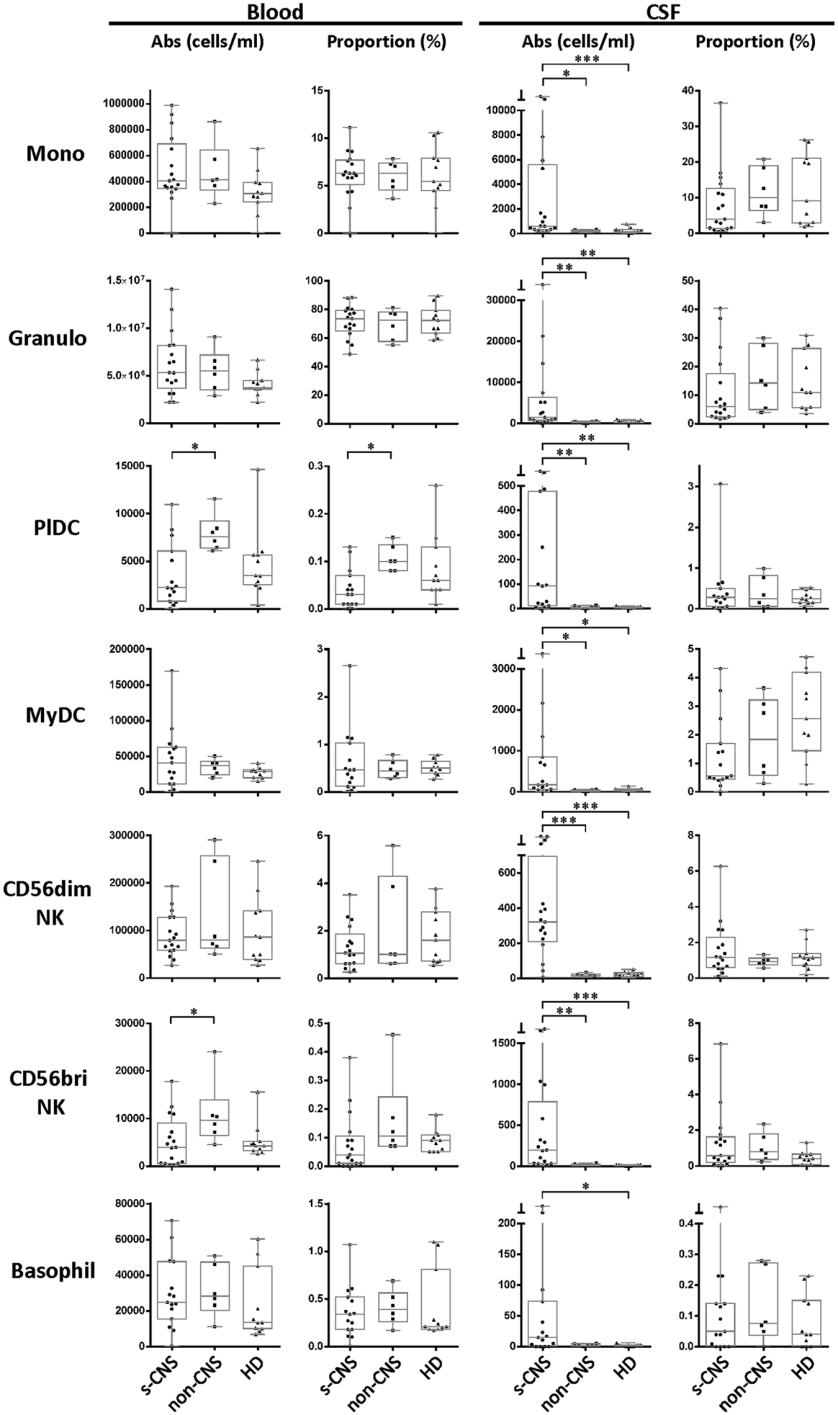
Immunophenotyping demonstrates increased absolute CSF cytotoxic (CD56dim) and immunoregulatory (CD56bright) NK cell populations, monocytes (Mono), and both myeloid (MyDC) and plasmacytoid (PlDC) dendritic cells in CSF of s-CNS patients. Absolute numbers and proportions of cell types were quantified by flow cytometry in CSF and blood as described in the Materials and Methods section in patients with s-CNS disease (N = 17), non-CNS disease (n = 6), and healthy donors (HD; N = 11). Proportions are calculated from the total CD45+ cells. *0.01≤p<0.05; **0.001≤p<0.01; ***0.0001≤p<0.001.

Twelve color immunophenotyping panels quantified absolute numbers and proportions of 12 major subtypes of immune cells in the blood and CSF [26] in the 17 s-CNS patients and were compared to 6 patients with previous fungal exposure leading to non-CNS disease as well as 11 HDs. These studies showed that s-CNS patients (Figs 2 and 3, right panels) had dramatic CSF elevations in absolute numbers of all components of the innate and adaptive immune responses as compared to non-CNS infected patients and HDs. Specifically, we observed significantly increased absolute counts of HLA-DR activated CD4+ and CD8+ T lymphocytes and B lymphocytes in the CSF of s-CNS compared with non-CNS and HD (Fig 2). We also identified significantly increased cytotoxic (CD56dim) and immunoregulatory (CD56bright) NK cells, monocytes, and myeloid dendritic cells (MyDCs) in the CSF of s-CNS cases compared with non-CNS cases and HD. Interestingly, there was a mild proportional decrease—albeit non-significant—in monocytes and MyDCs in the CSF of s-CNS cases compared with non-CNS cases and HD (Fig 3). Elevations in absolute granulocyte counts, attributed to steroid therapy in some patients, were the other CSF abnormality that differentiated meningitis patients from non-CNS patients and HD controls who did not receive steroids (Fig 3). However, no blood immunophenotyping differences were noted among the groups except for increased absolute counts and proportions of plasmacytoid dendritic cells (PlDCs) in the blood of non-CNS patients compared to s-CNS. Importantly, few CSF or blood immunophenotyping abnormalities were observed in patients with non-CNS disease compared to HDs, the latter likely due to the well-controlled nature of the disease and length of therapy.

In addition, CSF NFL levels were assayed, which are highly stable, neuron sensitive and specific biomarkers of axonal damage that fill the axon core and are released during neuronal damage [27–29]. Interestingly, we found a more than 10-fold, statistically significant increase in CSF NFL among s-CNS compared with non-CNS and with HD (S3 Fig). This suggests that the increased inflammatory response in s-CNS disease is not a benign or protective response, but is accompanied by elevated markers of neuronal damage.

### Assessment of Ag-specific T cells reveals compartmentalization of anti-cryptococcal Th1 T-cell immunity

Because we observed that cells of the adaptive immune system were elevated in the CSF of s-CNS patients to a similar or even greater degree than cells of innate immunity, we tested if these cells recognized cryptococcal antigens in a subgroup of 8 s-CNS patients, and if so, whether they secreted IFN-γ, which is thought to confer protective immunity. Five patients with non-CNS disease served as an appropriate comparator in this case, because they had been exposed to the cryptococcal fungus and therefore would be expected to have memory and effector T cell responses specific for cryptococcal antigens. Since it is known that only memory/effector T cells routinely cross the blood-brain barrier to perform immunosurveillance functions [30], intrathecal enrichment for cryptococcal-specific T cells have to be demonstrated against individuals with pre-existing cryptococcal exposure to be biologically meaningful.

Because the immunodominant epitopes of *Cryptococcus* have not been well defined and the s-CNS and non-CNS groups would require matching for major histocompatibility complex antigens (MHC) for peptide-based assays, we instead utilized a novel methodology based on the co-culture of autologous antigen (Ag)-loaded mature dendritic cells (mDCs) and T cells followed by flow cytometry-based confirmation of antigen-specificity as previously described [31]. In this assay, autologous DC’s phagocytose complex antigens (such as a cryptococcal homogenate) and select those epitopes that have the strongest binding to a patient’s MHC molecules, thus mimicking in vivo conditions as much as possible. Furthermore, this assay allows simultaneous read-outs of both CD4+ and CD8+ T cell responses, which is not possible in peptide-based assays. In addition to cryptococcal cell homogenate, we used a more defined mannoprotein (MP) preparation, which has been used previously in HIV-related cryptococcal infections to assess T-cell immune responses [11,32,33].

**Fig 4:**
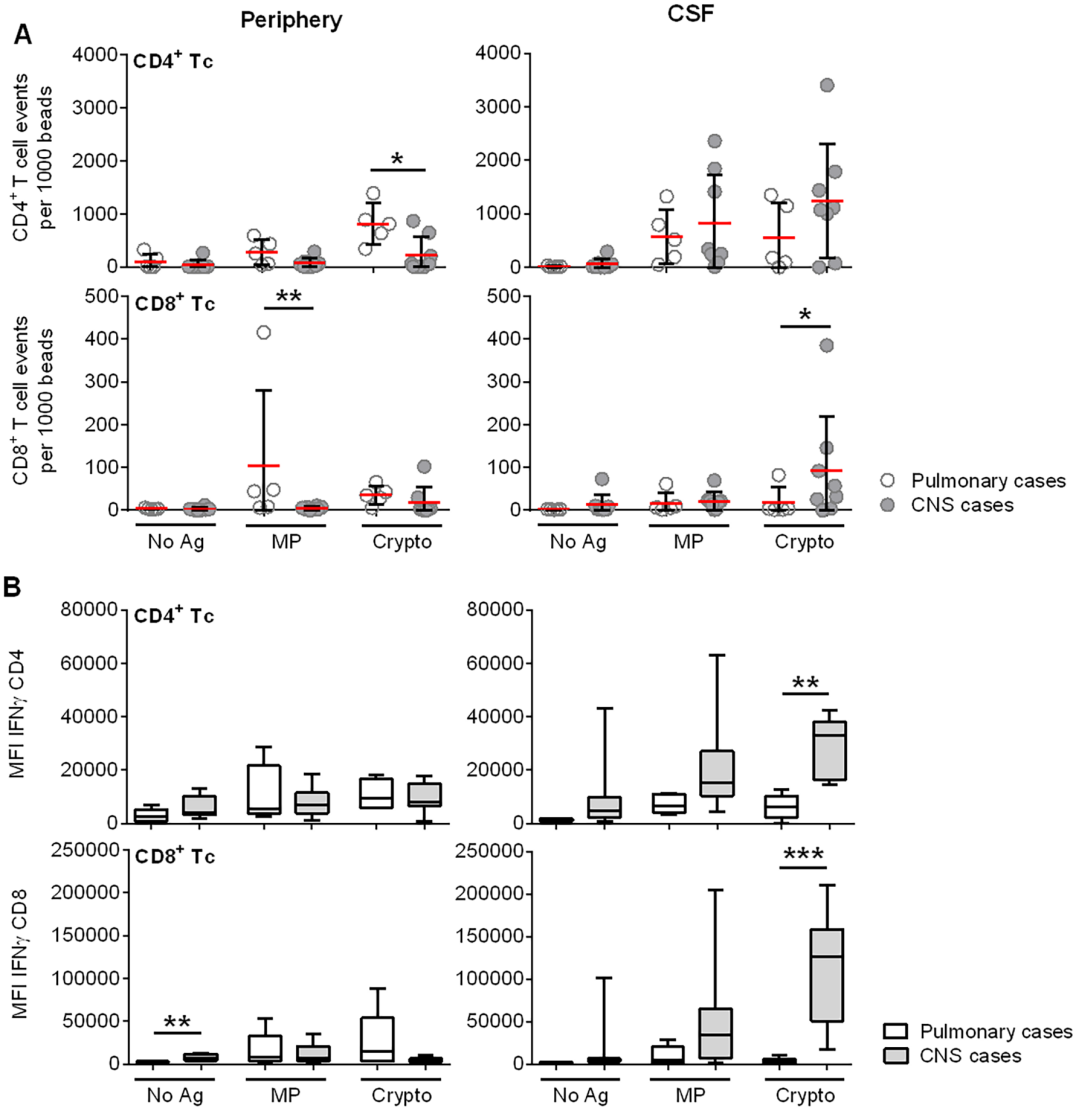
*Ex vivo* cryptococcal antigenic stimulation by pulsed autologous mature dendritic cells (mDCs) co-cultured with T lymphocytes from CSF or blood demonstrates compartmentalization of immune responses in a subgroup of 8 s-CNS and 5 non-CNS patients. (A) Sum of cytokine-producing IFN-γ+, TNF-α+, and IFN-γ+/TNF-α+ CD4+ and CD8+ T lymphocytes (after mDC presentation of MP and Crypto). Events are normalized to fluorescent beads. B) IFN-γ production of activated CD4+ and CD8+ T lymphocytes after Crypto presentation by mDCs. Tc = T lymphocyte; Crypto = glass bead-fractured, heat-killed *C*. *neoformans* strain H99; MP = *C*. *neoformans* mannoprotein; No Ag = no antigen (un-loaded mDCs). Open circles and bars are representative of non-CNS (Pulmonary) cases, filled circles and bars of s-CNS (CNS) cases. Error bars specify minimum to maximum values. *0.01≤p<0.05; **0.001≤p<0.01; ***0.0001≤p<0.001.

Using this approach, significant proliferative and secretory responses of CD4+ and CD8+ cells were exhibited in response to DCs loaded with either cryptococcal homogenate or purified cryptococcal MP. Higher proliferation of blood CD4+ T lymphocytes co-cultured with Ag-loaded DCs (Fig 4A, top left panel) was observed in the non-CNS patients compared with the s-CNS patients. In contrast, higher proliferation of CSF CD8+ T lymphocytes (Fig 4A, bottom right panel) was observed in the s-CNS patients after co-culture, suggesting a compartmentalization of the proliferative responses. Proliferation of CSF CD4+ cells was also observed in s-CNS patients compared to the unstimulated cells from the same patients, but was not statistically greater than the non-CNS patients in this small cohort of patients. In addition, increased IFN-γ secretion (Fig 4B, right panel) but not TNF-α was observed in both CD4+ and CD8+ T cells from CSF of s-CNS patients compared to those from non-CNS patients. These data suggest an ongoing compartmentalized antigen-specific Th1 response in the CSF.

Interestingly, responses to homogenate were greater than to that of the purified MP. MP was made from an acapsular strain (cap67) having serotype D background and serotype D is unusual in the U.S., whereas the homogenate was derived from H99, a serotype A strain—a clinical strain that is much more prevalent in the U.S.. In addition, MP is a purified component of the fungus, which undergoes mannosylation [34,35], but does not contain cell wall carbohydrates, lipids, or nucleic acids found in homogenates that could boost responses due to toll-like receptor (TLR) and c-lectin receptor (CLR) stimulation [36,37].

### CSF soluble biomarkers suggest a robust Th1 response within the intrathecal compartment of s-CNS patients

**Fig 5:**
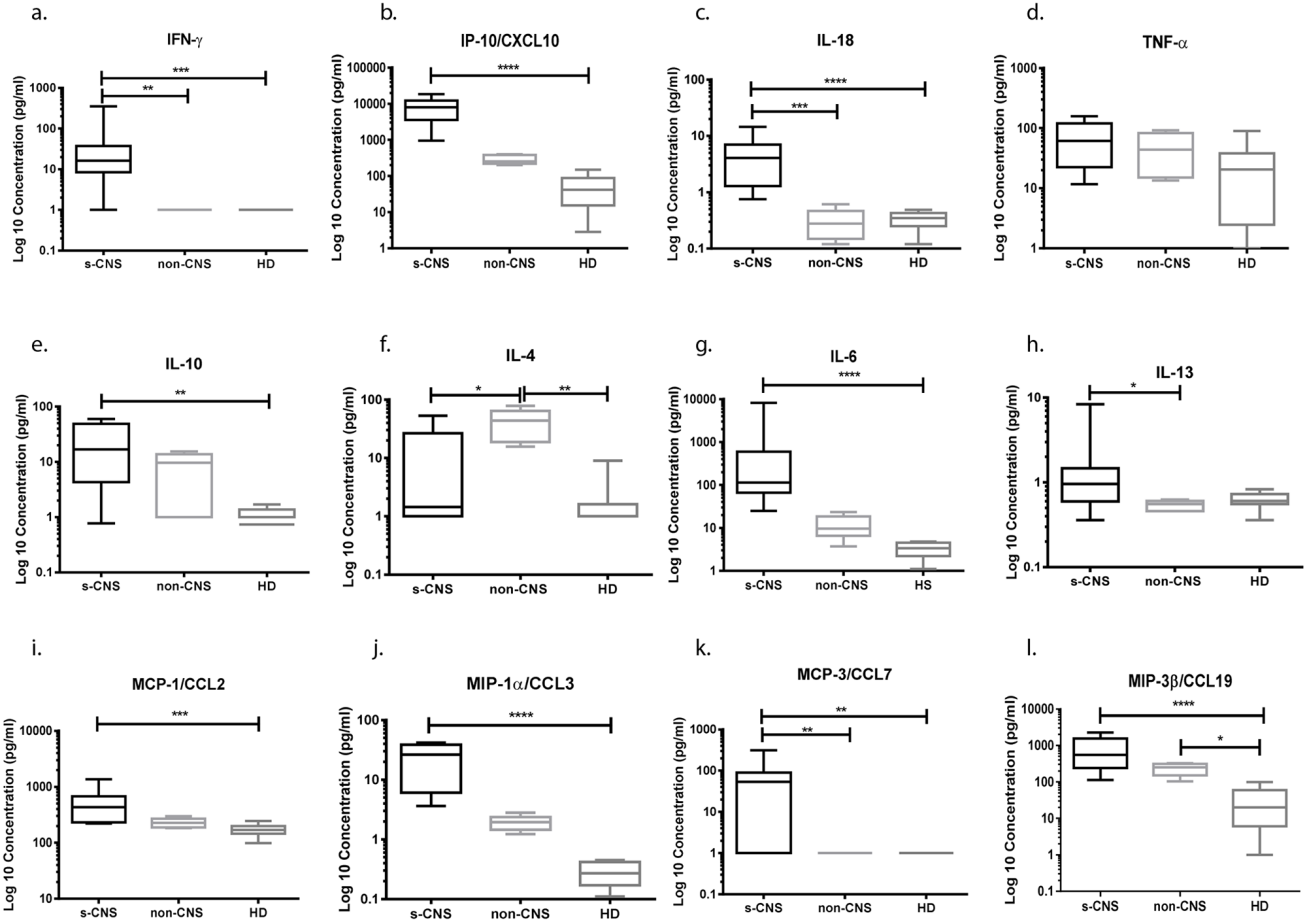
Intrathecal cytokine and chemokine profiles reveal elevated IFN-γ and related inflammatory responses in cryptococcosis patients with s-CNS disease compared with non-CNS disease and healthy donors (HD). CSF was analyzed using Luminex assays according to the Materials and Methods section. (a) IFN-γ, (b) CXCL10 (IP-10), (c) IL-18, (d) TNF-α, (e) IL-10, (f) IL-4, (g) IL-6, (h) IL-13, (i) MCP-1/CCL2, (j) MIP-1α/CCL3, (k) MCP-3/CCL7, and (l) MIP-3β/CCL19. Cytokine and chemokine concentrations were expressed as log_10_ picogram per milliliter (pg/ml) in s-CNS and non-CNS patients and HD. IL-8, GM-CSF, M-CSF, IL-12p40, IL-17, and IFN-α2 are not shown (without statistically significant differences among case groups). Error bars represent minimum to maximum values. *0.01≤p<0.05; **0.001≤p<0.01; ***0.0001≤p<0.001; ****p<0.0001.

To further assess CSF functional responses, expression of representative soluble cytokine and chemokines was determined among patients at the time of the cellular studies described in Fig 3. CSF from s-CNS patients demonstrated significantly increased intrathecal production of soluble IFN-γ, as well as interferon-induced IL-18, and chemokines CXCL10, as compared to the non-CNS patients or HD (Fig 5a–5c). Although IL-6 induces c-reactive protein (CRP) in the liver via the JAK/STAT pathways [38], no correlation was noted between serum CRP and CSF IL-6 and there was no difference between serum CRP of CNS vs. non-CNS cases (Table 1). The pro-inflammatory IL-6 (Fig 5g) similarly demonstrated increased expression in CSF of s-CNS patients as was the cytokine, IL-10 (Fig 5e). Interestingly, TNF-α levels were not significantly elevated (Fig 5d), consistent with the observed lack of increased TNF-α cellular production by T-cells incubated with ex vivo antigen-stimulated DCs described in the previous section and/or a functionally insufficient immune response from monocytes. S-CNS patients also produced significantly elevated levels of the chemokines CCL2 (monocyte chemoattractant protein-3 [MCP-1], CCL3 (macrophage inflammatory protein-1α [MIP-1α]), CCL7 (monocyte chemoattractant protein-3 [MCP-3]), and CCL19 (macrophage inflammatory protein-3β [MIP-3β]), compared to HD (Fig 5i–5l). Since these chemokine ligands are chemotactic for monocytes and T-lymphocytes, but CXCL10 is chemotactic only for CXCR3+ T-lymphocytes, one can infer the relative infiltration of monocytes compared to T-cells in the CSF based on the ratio of CCL3/CXCL10, for example. Statistically significant decreased CCL2/CXCL10 and CCL3/CXCL10 among s-CNS compared with HD and CCL19/CXCL10 among s-CNS compared with non-CNS and with HD corroborated decreased circulating monocyte CSF populations vis à vis T lymphocyte trafficking to the CSF (S2 Fig). In addition, non-CNS patients had significantly more detectable intrathecal IL-4 than s-CNS and HD (Fig 5f). These data again suggest a robust Th1-polarized response with evidence of active inflammatory activation within the intrathecal compartment.

### Brain Immunohistochemistry in 2 brain biopsies and an autopsy series of non-HIV-related patients with s-CNS disease demonstrates a predominance of tissue macrophages accompanied by T lymphocytes

**Fig 6:**
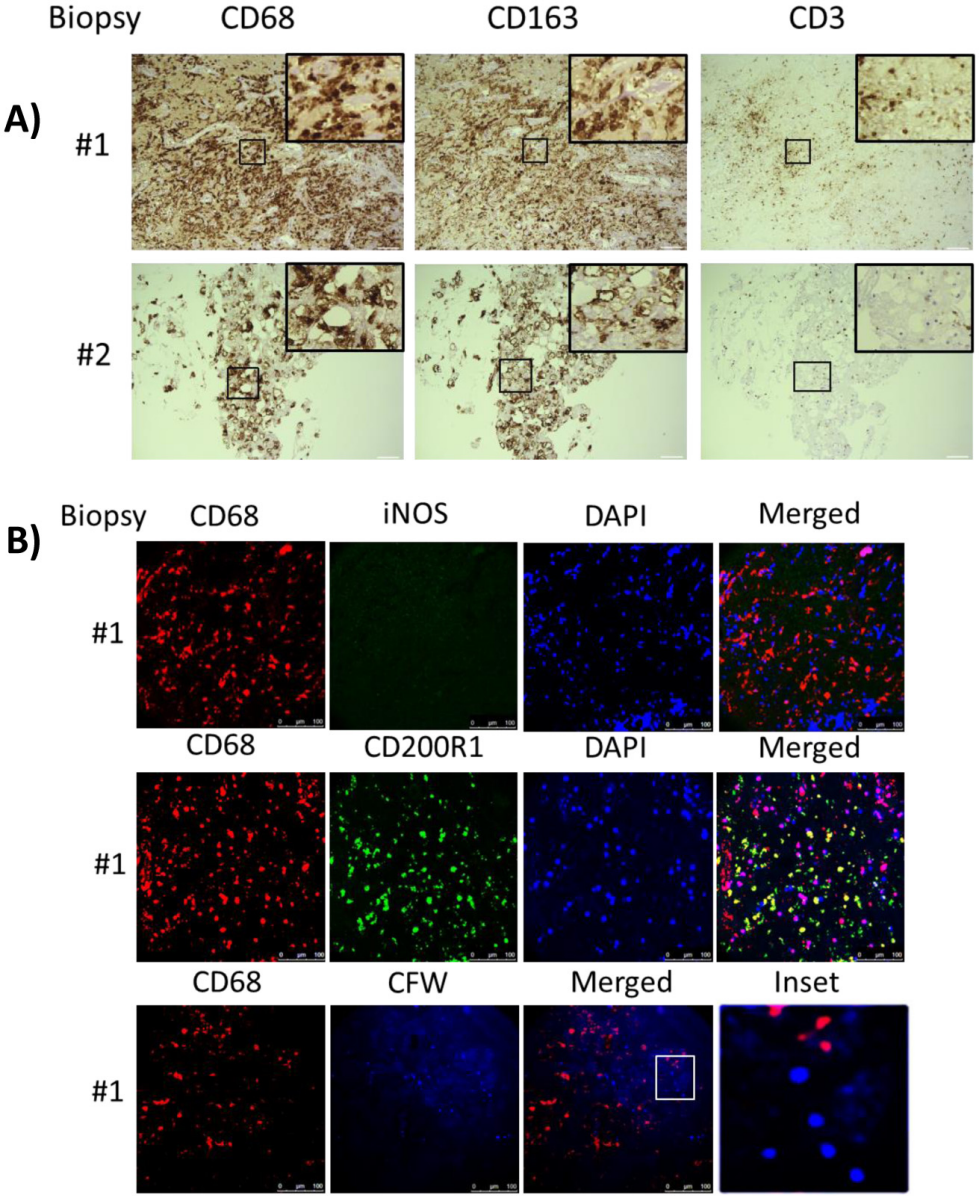
Brain biopsy specimens from patients with s-CNS cryptococcosis demonstrate macrophage and T-cell tissue infiltration. A) Diagnostic specimens obtained from two s-CNS patients were stained with macrophage markers CD 68 and CD163 and T-cell marker CD3. B) Immunofluorescence of brain biopsy of one patient after staining with macrophage marker CD68 and M1 marker (iNOS) and M2 marker (CD200R1), fungal stain, calcofluor white (CFW) and nuclear stain, 4',6-diamidino-2-phenylindole (DAPI). Magnification at 10 x with inset at 20x and scale bar set at 100 μm for 10x images.

Analysis of the intrathecal compartment by CSF sampling may be limited due to differences between CSF circulating and tissue-penetrant cells. Thus, brain biopsies obtained from two of the s-CNS patients as part of their diagnostic workup were examined using CD68, CD163, CD3, CD4 and CD8 staining. These biopsies were done prior to steroid therapy. Both biopsies demonstrated a predominance of CD68 positive monocyte/macrophages as well as the presence of T-cell infiltration in the region of the biopsy (Fig 6), suggesting that reductions in CSF circulating monocytes may be due to tissue infiltration of this cell population in these patients. Interestingly, while macrophages underwent tissue infiltration, iNOS and CD200R1 staining in one of the biopsy specimens that contained sufficient tissue for more detailed analysis suggested the macrophages underwent a non-protective alternative activation and less than 1% of fungal cells were observed to be co-localized with macrophages, suggesting poor phagocytic function during cryptococcal brain infection of this patient (Fig 6B).

**Fig 7:**
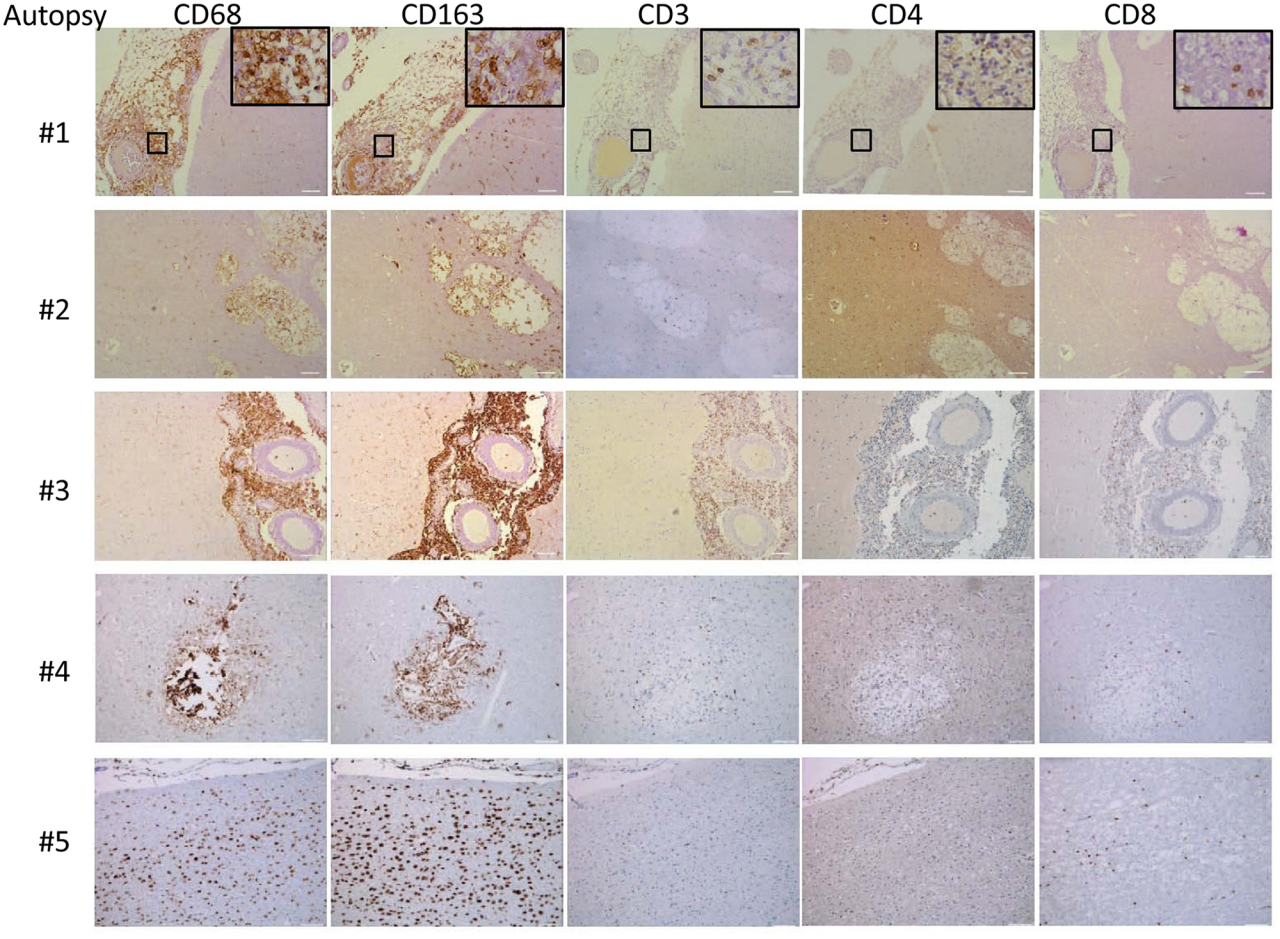
Brain immunohistochemistry staining of brain autopsy specimens from patients who died of s-CNS cryptococcosis demonstrate macrophage and T-cell tissue infiltration. Autopsy specimens from meninges (#1, #2), Virchow-Robin space (#3, #4) or within brain parenchyma (#2, #5) were stained with macrophage markers CD 68 and CD163 and T-cell markers CD3, CD4 and CD8. Magnification at 10x with inset at 20x and scale bar set at 100 μm for 10x images.

Because of the limited availability of brain tissue in the live s-CNS cohort, an analysis of NIH clinical center records (1957–2009) was conducted to identify non-AIDS infected patients who died of cryptococcal meningitis by autopsy (Table 2). All patients were treated with amphotericin-based regimens but two had had Hodgkin’s disease. Review of the autopsy report allowed exclusion of patients with known comorbidities such as sarcoidosis, HIV or other co-infections and inclusion of patients who died with cerebral herniation. During his neurological decline, one patient also developed bronchopneumonia from aspiration. Characteristics of the five s-CNS patients who died of cryptococcal meningoencephalitis are listed in Table 2. In these patients, immunohistochemistry (IHC) showed extensive leptomeningeal and Virchow-Robin space infiltration of CD68 and CD163 macrophages as well as the presence of CD3, CD4, and CD8 T lymphocytes (Fig 7).

**Table 2:** Autopsy report summary of patients who died of severe CNS (s-CNS) cryptococcosis.

	Autopsy #	1	2	3	4	5
	**Age (years), Race, Sex**	61y, W, F	21y,W, M	59y, W, F	35y, W, M	26y, W, F
	**Time from Symptoms to Death**	7 months	4 months	6 months	3 months	16.5 months
	**Time from Treatment to Death**	1 week	7 weeks	2 weeks	2 months	2.5 months
	**CAUSE OF DEATH**	respiratory failure due to bilateral bronchopneumonia	decerebrate; grand mal seizures	respiratory failure due to brain compression	respiratory failure due to CNS depression	respiratory failure
**TERMINAL EVENT**	**Coma**	frequent confusion with complete disorientation;	drowsy, obtunded	noxious stimuli withdrawal only	obtunded	no
	**Brainstem herniation signs**	Cheyne-Stokes respirations; small divergent nonreactive pupils	dilated pupils; decerebrate posturing; hyperventilation	dilated pupils—> deviation to left	Cheyne-Stokes respirations	none
	**Deep tendon reflexes (DTR); Babinski's sign**	hyperreactive DTR; absent Babinski	normal DTR; Babinski present bilaterally	hyperreactive DTR; Babinski present bilaterally	hyperreactive DTR; absent Babinski	normal DTR; absent Babinki
	**Spinal cord compression sign**	right hemiparesis	left hemiparesis; incontinence	incontinence	clonus	none
	**Cranial Nerve (CN) Palsy**	mild CN V	left CN VI	left CN VI, VII	mild left CN VII	none
	**Other complications**	right arm seizure	papilledema	papilledema	papilledema with retinal hemorrhages	papilledema; left middle and posterotemporal lobe slowing on EEG
**LP FINDINGS WITHIN ONE WEEK OF DEATH**	**Spinal fluid opening pressure (mmH20)**	330	270	300	330	120
	**Protein (mg/dl)**	140	111	110	45	148
	**Glucose (mg/dl)**	15	15	12	20	28
	**Cell count**	11 (PMN predominant)	18	808 (403 PMN, 398 lymphs)	8	0
	**India ink**	positive	positive	positive	positive	negative
	Culture (Cryptococcus neoformans*)	positive	positive	positive	positive	positive
	**CONCOMITANT ILLNESS**	Gastric ulcer; Dumping syndrome post partial gastrectomy; aspiration pneumonia	Hodgkin's lymphoma^1^	Diabetes mellitus	Hodgkin's lymphoma^1^	pneumonia; pulmonary fibrosis
	**TREATMENT**	Amphotericin B; no shunt	Amphotericin B; Prednisone 1mg/kg/d**; no shunt	Amphotericin B; emergency decompression with right lateral ventricle catheter	Amphotericin B; Prednisone 1mg/kg/d**	Amphotericin B; Prednisone 1mg/kg/d**

Cryptococcus autopsy cases for brain immunhistochemistry that have been collated according to the following inclusion criteria based on findings 1 week prior to death: (1.) cryptococcal meningoencephalitis with or without dissemination (CSF India ink OR culture positive), (2.) Glasgow coma score < 8 for > 48 hours, (3.) presence of Babinski or other upper motor neuron signs OR seizure activity, and (4.) exclusion of competing risks.

PMN = polymorphonuclear leukocytes

*PCR of our paraffin embedded brain autopsy specimens revealed *C*. *neoformans* in all 5 autopsy specimens.

**tapered off by time of death

^1^ in remission

**Fig 8:**
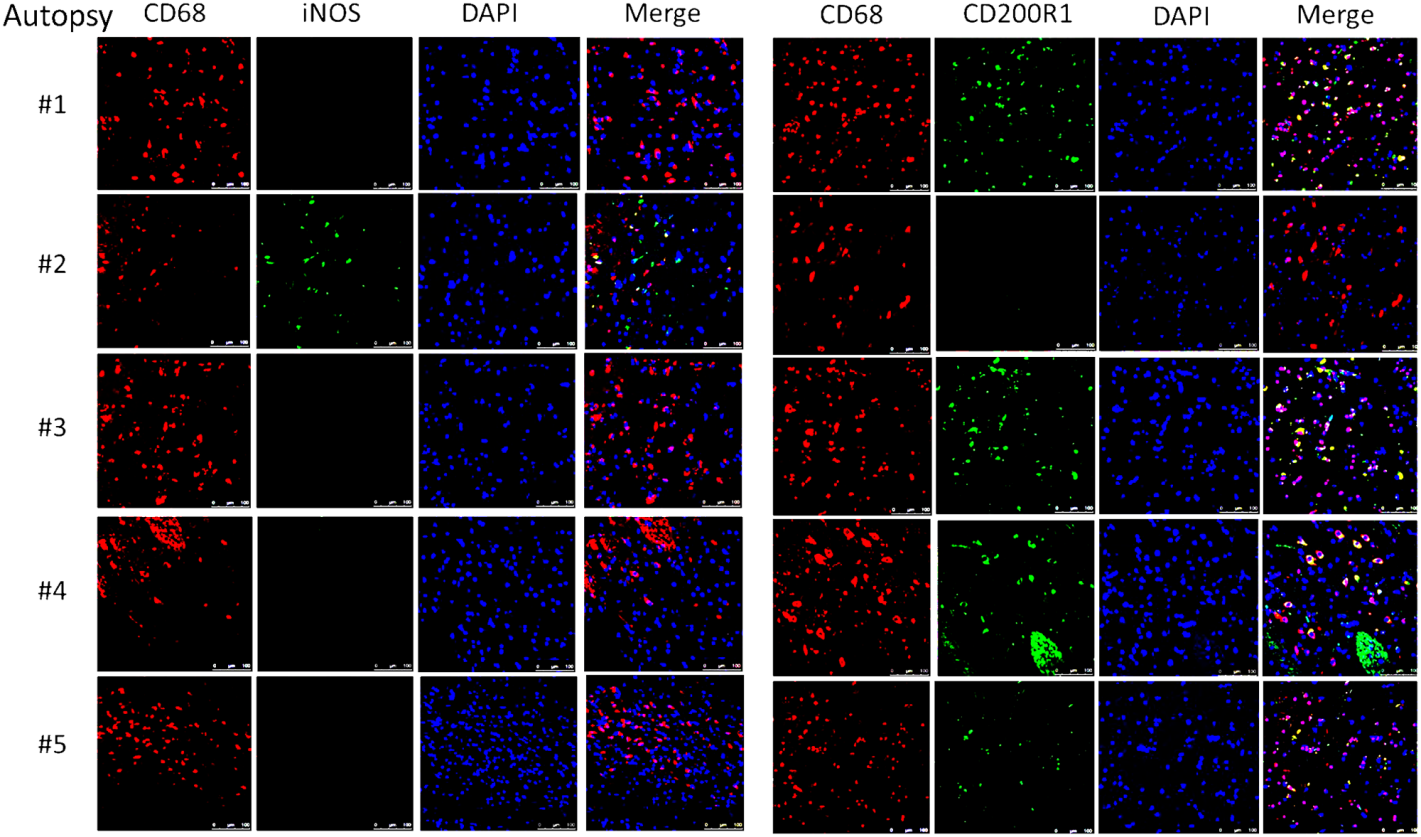
Brain immunofluorescence of autopsy specimens from patients who died of s-CNS cryptococcosis demonstrates macrophage and T-cell tissue infiltration. Fluorescent macrophage markers (CD68) and M1 marker (iNOS) and M2 marker (CD200R1) were used to stain cells within the peri-vascular brain parenchyma. DAPI was used for nuclear localization. Magnification at 10x with inset and scale bar set at 100 μm for 10x images.

**Fig 9:**
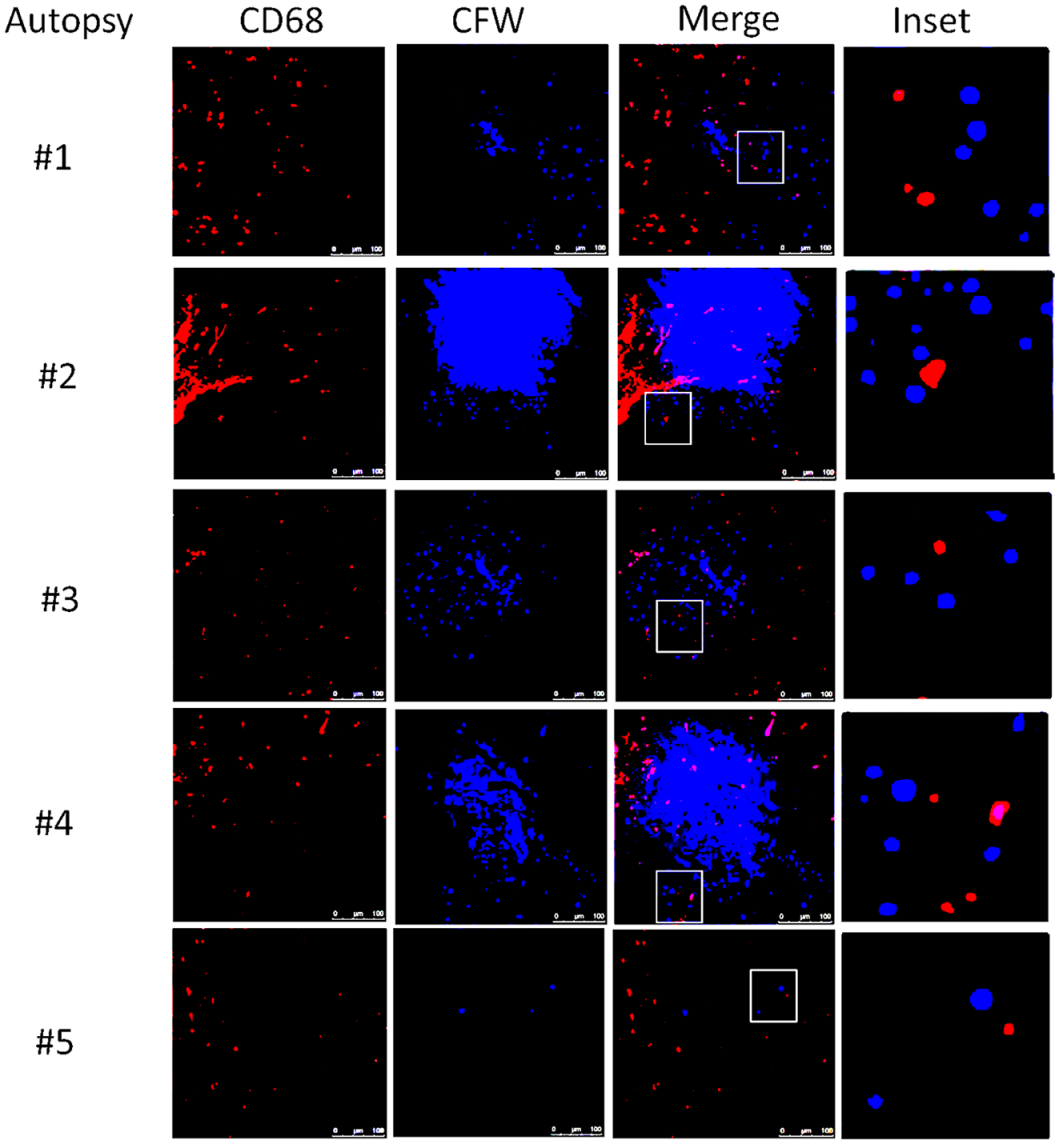
Brain immunofluorescence of autopsy specimens from patients who died of s-CNS cryptococcosis. Fluorescent macrophage marker (CD68) and fungal marker calcofluor white (CFW) was used to stain cells within the peri-vascular brain parenchyma. Magnification at 10x with inset and scale bar set at 100 μm for 10x images.

This suggests that the apparent decrease of myeloid cells in the CSF obtained in the present cohort may have been due to relative increases in monocyte/DC infiltration of the brain parenchyma. This would appear to be an effective monocyte response. However, further analysis of the autopsy specimens using fluorescent probes of tissue sections (Fig 8) indicated that the majority of the patients (4/5) demonstrated an M2 non-protective bias (CD200R1+) to their CD68 positive monocyte/macrophages and calcofluor white fungal staining (Fig 9) demonstrated poor uptake of fungal cells (< 1%) by these macrophage populations, regardless of the monocyte activation status, consistent with the result in the biopsy specimen.

## Discussion

The present study sought to comprehensively investigate innate and antigen-specific intrathecal immunity of HIV-uninfected individuals with clinically severe, cryptococcal meningoencephalitis to guide treatment strategies of a disease group with high mortality. These studies were prompted by several potential conflicting issues in human cryptococcal disease including 1) a presumed T-cell defect in previously healthy patients based on paradigms developed with HIV-associated infections, 2) the unknown contributions of microbiological control vs. dysfunctional/paradoxical immune responses in CNS disease and 3) the resultant ambiguities as to whether microbiological control should be intensified in severe cases with antifungals or augmentative cytokines such as IFN-γ or adjunctive immune suppression with steroids or other agents. A dramatic clinical and radiological response in two patients to steroids further provided a rationale for these studies, focusing on subsequent, consecutive patients recruited to the NIH clinical center with CNS cryptococcosis. Likely due to referral bias, all 17 were severely affected with altered mental status, suggesting a label of s-CNS disease to indicated the severity of the cohort. The clinical process appears to be quite long lasting, with clinical severity and MRI brain findings following the clearance of cryptococcal antigen over many months or a year as described in S1 Fig.

Interestingly, despite an expectation of low T-cell function, this cohort of s-CNS disease, displayed intrathecal expansion and activation of cells of both innate and adaptive immunity including HLA-DR+ CD4+ and CD8+ cells and NK cells. These expanded CSF T cells were enriched for cryptococcal-antigen specific CD4+ and CD8+ T cells that are critical in protection from the fungus [39], secreting the protective cytokine, IFN-γ [40] as well as a lack of highly elevated CSF levels of typical T-cell specific Th2 cytokines IL-4 and IL-13 (IL13 was barely significant)[41], or typical Th17 cytokines IL-17 or GM-CSF (also not shown). Animal studies have suggested that IL-17 is not required for classical activation of macrophages in cryptococcal infections [42] and may not be as important as Th1-type responses [43]. Because patients with s-CNS had more than 1000-fold increased numbers of CSF T cells, in comparison to systemic *Cryptococcus* disease controls (i.e., non-CNS) and HDs, lack of prototypic Th2 and Th17 cytokines in the CSF of these patients is highly informative and in our opinion represents strong evidence for Th1 skewing of infiltrating T cells. This Th1-biased response was unexpected as such a response is thought to be protective against *C*. *neoformans* in both mouse studies [44] and human experience where T-cell defects from HIV/AIDS [11] or during solid organ transplant conditioning [45], predispose to cryptococcal infections. Interestingly, Jarvis *et* al noted different antigen-specific functional T cell phenotypes in HIV-infected patients with cryptococcal meningitis and another intracellular pathogen—tuberculosis [11]. These represent important findings and suggests that the clinical worsening in Patient 1 (Fig 1) after recombinant IFN-γ therapy may have been due to exacerbation of this paradoxical inflammatory state and suggests caution in using this suggested approach [9,10] in non-HIV-related s-CNS cryptococcal disease. Indeed, we found that this inflammatory response was accompanied by a more than 10-fold elevation in CSF NFL, a biomarker of axonal damage in diverse neurological disease [27,28] and suggests that the paradoxical inflammatory response results in ongoing neurological damage. Thus, the present study represents a critical application to human disease whereby too much of an immune response can be detrimental by virtue of host-induced damage [15,16].

Interestingly, we observed this aggravated immune response in patients infected with either *C*. *neoformans* or *C*. *gattii*, the latter of which is reported to cause increased inflammatory responses in mice [46] and human patients [14]. This paradoxical immune response in non-HIV-infected individuals bears resemblance to that of cIRIS in HIV/AIDS that occurs after initiation of anti-retroviral therapy (ART). In cIRIS, patients who initially show a clinical and microbiological response to anti-fungal therapy develop, after initiation of ART, clinical worsening of meningitis symptoms including deteriorations in mental status or increased lymphadenopathy, cutaneous lesions or pulmonary nodules, despite continued negative fungal cultures [47]. Immunologic studies of HIV-related cIRIS identified within the blood compartment, a similar Th1-driven response consisting of enrichment of CD4+ and CD8+ T cells coexpressing activation markers CCR5 and CXCR3 with detection of IFN-γ-dependent chemokines such as CXCL10 [48]. Within the CSF compartment, cIRIS is associated with elevations of IFN-γ, TNF-α, G-CSF, VEGF, and eotaxin (CCL11) [49,50]. The paradoxical response is thought to be due to a reversal of HIV-associated immune defects in the presence of continued fungal antigen tissue burden [51].

However, a predominant difference between cIRIS and the intrathecal inflammatory response of s-CNS patients in the present study may lie in the M2 skewing of CNS-tissue infiltrating macrophages in the latter group which is a non-protective response in animal models of cryptococcosis [52]. This M2 phenotype was suggested by the predominance of CD68+, CD200R1+ monocytes found in our autopsy and biopsy marker studies and was also corroborated by a lack of increased CSF levels of IL-12p40 and TNF-α as well as elevated CSF levels of IL-10 in the s-CNS patients. This M2 skewing was present regardless of the presence of steroids and was present early in the course of the disease as demonstrated in the diagnostic biopsy #1. It is unclear whether the predominant intrathecal cellular source of these differentially-expressed cytokines are T cells or monocytes/macrophages, which are traditionally robust sources of TNF-α and IL-10 [53]. Given the Th1 bias of T cells in the present study, the tissue infiltrating M2 cells are a potential source of IL-10 and the M2 bias likely reduced production of fungal protective TNF-α [54,55]. This latter defect may also explain the critical lack of fevers in these patients, which is a major factor leading to diagnostic delays in this patient population.

A second main difference of the non-HIV response from cIRIS is that no immunostimulatory or immune restoring interventions were required to produce the paradoxical immune response in the HIV-uninfected individuals. The fact that clinical deterioration in the present series occurred, usually after a month of fungicidal therapy and with negative CSF cultures suggests that liberation of fungal antigens during therapy may have induced intrathecal activation of antigen-specific T cells. Indeed, intact *C*. *neoformans* cells prior to therapy are protected from immune recognition by an immune suppressive polysaccharide capsule [56], an extracellular laccase [57] and components of the fungal cell wall [58], lysis of which results in release of intracellular fungal antigens.

Because the presented functional studies are extremely laborious, require large amounts of fresh biological samples and the targeted patient population is rare and includes very sick subjects, a natural limitation of our study is its small sample size and limited sample numbers per patient. In addition, the variability in treatment duration and steroid use for cerebral edema may make heterogeneity a source of concern, though the significant findings despite this are noteworthy. The present study was thus an important first step in the description of the immune response in this population, but did not attempt to use specific immune responses to stratify outcomes or follow the evolution of the disease, although elevated Th1 responses were identified over a prolonged time frame as long as symptoms and antigen persisted. The limitation of the patient population to those previously healthy helped to add a measure of uniformity to the population, but may limit the application to more immunosuppressed patients such as those undergoing transplant conditioning. In addition, referral bias likely led to a higher disease severity that may not be applicable all previously healthy patients with CNS disease. However, the expected high mortality of this group makes its study highly compelling for suggesting studies to improve therapy. In addition, it is important to note that the results here represent patients that deteriorate during effective fungicidal therapy and should not be applied to clinical issues on initial presentation when microbial control may be more important.

Despite the robust Th1 bias and inflammatory response, the immune response appeared to be ineffective to prevent or clear the fungus. Recent data demonstrating the relative rarity of non-HIV cryptococcal disease [5] despite high rates of exposure [59] also support a defective host response. Data acquired in the present study all point towards a defective innate immunity, and, more specifically, defects of the myeloid lineage. We identified poor proportional monocyte recruitment to the circulating pool of the CSF cells and a predominant non-protective M2 response in most of the autopsy specimens, as well as an inability to phagocytose the fungus in situ. This is consistent with our recent data demonstrating the presence of a STAT5 phosphorylation-blocking GM-CSF autoantibody in a subset of patients with cryptococcal disease [25,60] and suggest that future targeted studies should focus on the mechanistic assessment of cells of myeloid lineage, including evaluation of phagocytosis [61], oxidative burst [62]and antigen processing [63] important to cryptococcal defense. In conclusion, the present study suggests that an ineffective macrophage response in the setting of intact T-cell activation has the potential to cause increased susceptibility of the patient to the fungus and severe CNS disease after initiation of effective anti-fungal therapy. We infer that there may be either intrinsic or extrinsic defects at the immune synapse between Th1 cells and antigen presenting monocytes or further downstream that lead to a paradoxical M2 polarization. These studies also provide pathophysiological support for studies of adjunctive immunosuppressive trials in severe non-HIV related cryptococcal disease to control paradoxical immune responses after sustained microbiological control in a CNS fungal disease with high mortality.

## Materials and Methods

### Ethics statement

The National Institute of Allergy and Infectious Diseases (NIAID) Institutional Review Board (IRB) approved this study under NIAID Protocol #93-I-0106. The National Institute of Neurological Disorders and Stroke (NINDS) approved the study under NINDS Protocol #09-N-0032. All subjects provided written informed consent directly or via their durable power of attorney, after obtaining National Institutes of Health (NIH) Bioethics consultation.

### Participants and patient clinical data

We studied 17 consecutive patients with cryptococcal meningitis who were all found to exhibit characteristics of severe CNS cryptococcal disease (s-CNS) from an ongoing recruitment cohort of 80 patients with cryptococcal disease in previously healthy individuals without known immunocompromising conditions (HIV 1/2, HTLV-1, and Hepatitis B and C seronegative individuals, idiopathic CD4 lymphopenia (ICL), those without antecedent known malignancy, liver/renal failure, sarcoidosis, systemic autoimmune disorders, and recipients of transplants or immunosuppressive medications such as corticosteroids, calcineurin inhibitors, etc…) S-CNS was defined as persistent or deteriorating mental status changes (Glasgow Coma Score, GCS < 15) despite adequate amphotericin B-based antifungal therapy (≥6 weeks) and all had repeated negative CSF cultures after 6 weeks of therapy. Comparator groups were selected as patients within the larger cohort with non-CNS disease including 5 with pulmonary and 1 with isolated spinal bone disease and 11 healthy donors (HD). Diagnosis was made by CSF India ink stain and/or culture, or a positive mucicarmine stain and/or culture from a biopsy site or a positive cryptococcal antigen testing (Meridian Cryptococcal Latex Agglutination System; Meridian Bioscience), according to previous case definitions [64]. We performed an extensive chart review of 17 s-CNS cases and 6 non-CNS cases and tabulated pertinent clinical information related to the timeline of their infection in Table 1. Mental status was evaluated as Glasgow Coma Score (GCS) by clinical staff of the NIH clinical center during 24 h monitoring, using a standardized protocol and expressed as a 10 day average +/- SEM of all measurements. (http://www.commondataelements.ninds.nih.gov/Stroke.aspx#tab=Data_Standards). Evaluations were performed 3–4 times per day between the hours of 8 am and 11 pm.

### Ex vivo peripheral blood and CSF immunophenotyping

Immunophenotyping of peripheral blood cells was performed within 60 min of ex vivo collection on anticoagulated blood after osmotic lysis of erythrocytes. CSF samples were placed on ice immediately after collection. Within 15 min of collection, the CSF (usually 20 ml) was spun and cell pellets were resuspended in 400 ml ice-cold X-Vivo media (Lonza, Alpharetta, GA). The Fc receptors of cells were blocked by using 2% immune serum globulin to prevent non-specific activation. A 12-color immunophenotyping panel to distinguish 12 leukocyte cell types was adapted using the markers in S1 Table from that previously described [26].

### Isolation of peripheral blood mononuclear cells (PBMCs) and generation of dendritic cells (DCs)

120 ml of heparinized blood samples were collected eight days before the scheduled lumbar puncture (LP). Isolation of PBMCs and generation of DCs with GM-CSF, IL-4 and 5% human serum were performed as described previously [31]. On day six of culture, immature DCs were co-incubated overnight with the following antigens (Ag’s) and subsequently stimulated for 48 hours: mannoprotein (MP), prepared as described [32], and cryptococcal heat-killed suspension (Crypto; both 5 μg/ml) with maturation factors, TNF-α, IL-1B, IL-6 and PGE2. Cryptococcal heat killed suspension was prepared by glass-bead fractured *C*. *neoformans* H99 strain ATCC 208821 ( = CBS8710) that had been heated at 90°C for 30 min and the suspension allowed to settle overnight at room temperature. The suspended material was recovered and quantified by protein analysis by using a BioRad assay according the manufacturer’s directions (BioRad, Hercules, CA). Optimal Ag-concentrations were determined in preliminary experiments by titration (i.e. inducing T cell proliferation without evidence of toxicity).

### Co-culture of Ag-loaded mature dendritic cells (s) and T cells

At the day of LP (day eight of culture) peripheral T cells were purified from frozen PBMCs by negative selection (Miltenyi Biotech, San Diego, CA). Peripheral and CSF T cells were then cultured in round bottom 96 well plates with autologous Ag-loaded mDCs (3x10^3^ mDCs: 3x10^3^ T cells) in a total volume of 100 μl X-Vivo media (Lonza) at 37°C, 5% CO_2_. After seven days of proliferation, T cells were re-stimulated overnight for the intracellular cytokine staining (ICCS) with twice as much newly differentiated, identically loaded autologous mDCs as used for the first co-culture in presence of Brefeldin A and monensin (eBioscience). Ag-specificity was detected by flow cytometric analysis of cytokine-producing CD4+ and CD8+ T cells.

### Flow cytometric analysis of surface markers and intracellular cytokines

T cells were analyzed for the surface markers CD3 (UCHT1), CD4 (RPA-T4), and CD8 (SK1), and intracellular expression of GM-CSF (GM2F3), IL-17 (eBio64DEC17), TNF-α (MAb11) and IFN-γ (B27; BD Biosciences, San Jose, CA and eBioscience). Data were analyzed with BD FACS Diva 6.1 (BD Biosciences).

Absolute numbers of cytokine-producing cells were determined by normalization with APC beads. For inter-patient comparison we standardized the number of cytokine positive events (i.e. IFN-γ^+^, TNF-α^+^, and double positive events) to 1000 beads each.

Mean fluorescence intensities (MFIs) of cytokine-secreting CD4^+^ and CD8^+^ T cells were determined by subtraction of the respective isotype controls. Negative values were adjusted to zero.

### CSF cytokine and NFL quantification

After lumber puncture, CSF samples were centrifuged at 300 g and stored at -80C until analysis. CSF from unstimulated CSF flow cytometry experiments on s-CNS, non-CNS, and HD above were analyzed using a sandwich-enzyme linked immunosorbent assay (Luminex, Bio-Rad). After including standards, we assayed for intrathecal evidence of IL-4, IL-6, IL-8/CXCL8, IL-10, IL-12p40, IL-13, IL-17, IL-18, IP-10/CXCL10, IFN-α2, IFN-γ, TNF-α, MCP-1/CCL2, MIP-1α/CCL3, MCP-3/CCL7, MIP-3β/CCL19, M-CSF, and GM-CSF. Detected amounts were expressed in log_10_ picogram/milliliter (pg/ml). CSF levels of NFL were measured according to the manufacturer’s instructions using a sandwich ELISA method (Uman Diagnostics AB; Umea Sweden).

### Brain immunohistochemistry and macrophage polarization studies

Brain immunohistochemistry (IHC) on five patients who died from s-CNS cryptococcal disease (and two biopsy specimens from live patients with s-CNS disease) were collated. The inclusion criteria for autopsies were based on findings one week prior to death: (1.) cryptococcal meningoencephalitis with or without extraneural infection (CSF India ink or culture positive and WBC > 50 and opening pressure > 200 mm); (2.) Glasgow Coma Score < 8 for > 48 hours; (3.) the presence of Babinski or other upper motor neuron signs or seizure activity; and (4.) exclusion of competing causes of death. Chromogenic staining using horse radish peroxidase-conjugated secondary antibody for T lymphocyte markers (CD3 [F7.2.38 clone], CD4, and CD8) and macrophage markers (CD68 [KP1 clone] and CD163) was performed (Dako North America, Carpenteria, CA) and representative images captured using Leica Software at 10x and 20x magnification. Polarization of macrophages was performed on autopsy and biopsy specimens using double immunofluorescence staining according the manufacturer’s directions (Abcam). M1 was characterized by CD68/iNOS expression and M2 was identified by CD68/CD200R1 co-staining as described [65]. Calcofluor white was used to stain cryptococcal cells and DAPI (4',6-diamidino-2-phenylindole) for nuclear staining. To detect cryptococcal DNA in the paraffin-embedded specimens, a DNA extraction was performed by using the blackPREP FFPE DNA Kit (Analytik Jena, Jena, Germany) with an additional lyticase treatment. Real-time PCRs were performed on the extracts to detect cryptococcal DNA, and specifically *C*. *gattii*, as described previously [66,67].

### Statistics

Ten day GCS averages were determined by averaging scores over successive 10 day intervals (N > 20 determinations), beginning on the first day of admission among each of two illustrated s-CNS patients. We used repeated measures ANOVA to account for the inter-correlated GCS values to determine statistical significant changes 10 days before and 10 days after corticosteroid therapy. For the CSF cytokine/chemokine analysis among s-CNS, non-CNS, and HD, the non-parametric Kruskal-Wallis test was utilized, controlling for multiple comparisons between each group using the Dunn procedure [68]. For NFL chain analysis, since we were interested in assessing only neuronal damage using s-CNS as the comparator, we performed a non-parametric Mann-U Whitney test adjusting for comparison between s-CNS vs. non-CNS and s-CNS vs. HD using a Bonferroni correction. CSF cytokine, chemokine, and NFL analysis was performed using PRISM (version 6.0, Graphpad Software, La Jolla, CA).

For immunophenotyping assays, one-way ANOVA (analysis of variance) was performed to explore the association of explanatory variable (diagnosis: s-CNS, non-CNS, and HDs) with 56 response variables which are the absolute numbers or proportions of adaptive and innate immune cells collected from CSF and blood staining samples. Since most of these response variables did not follow a normal distribution, Box-Cox transformation was applied (log- transformation for most of the variables) prior to ANOVA. Tukey’s method was used for pair-wise multiple comparisons. P-values were adjusted for multiple testing. A two sample t-test was used to test the difference between Ag-specific T cell proliferation and between MFIs in the non-CNS and s-CNS cohorts. Peripheral and intrathecal T cells were analyzed separately for CD4+ and CD8+ T lymphocytes. Zero MFI values were treated as missing values. Log transformation was applied to all outcome measures. These statistical analyses were performed using SAS version 9.3 (SAS Institute, Cary, NC) with p ≤0.05 considered a significant level.

## Supporting Information

S1 Fig Steroid-responsiveness of refractory cryptococcal meningitis in patient #2 from Fig 1. Top panel, Enhanced MRI T1 and FLAIR weighted images corresponding to hospital Day. Lower panels, indicated steroid dosage (black line) and CSF cryptococcal latex antigen titer (*).
(TIF)

S2 Fig CSF ratios of CCL2/CXCL10, CCL3/CXCL10, CCL7/CXCL10, and CCL19/CXCL10 (supplement to Fig 5). Depicted CSF chemokine ratios determined from data shown in Fig 5 of the CSF circulating pool of s-CNS patients compared with non-CNS patients and healthy donor controls (HD). Error bars represent minimum to maximum values. *0.01≤p<0.05; **0.001≤p<0.01; ***0.0001≤p<0.001; ****p<0.0001.
(TIF)

S3 Fig NFL analysis reveals significantly increased NFL levels in CSF of s-CNS compared with non-CNS or HD (supplement to Fig 5). CSF NFL levels assayed from CSF as described in Materials and Methods. *0.01≤p<0.05; **0.001≤p<0.01.
(TIF)

S1 Table Optimized combination of flourochrome-conjugated antibodies used to quantify 12 subpopulations of immune cells. (DOCX)
