# Supplementary figures and images for: Paradoxical Immune Responses in Non-HIV Cryptococcal Meningitis
Source: PLoS Pathog. 2015 May 28;11(5):e1004884. doi: 10.1371/journal.ppat.1004884 (PMC4447450; doi:10.1371/journal.ppat.1004884)

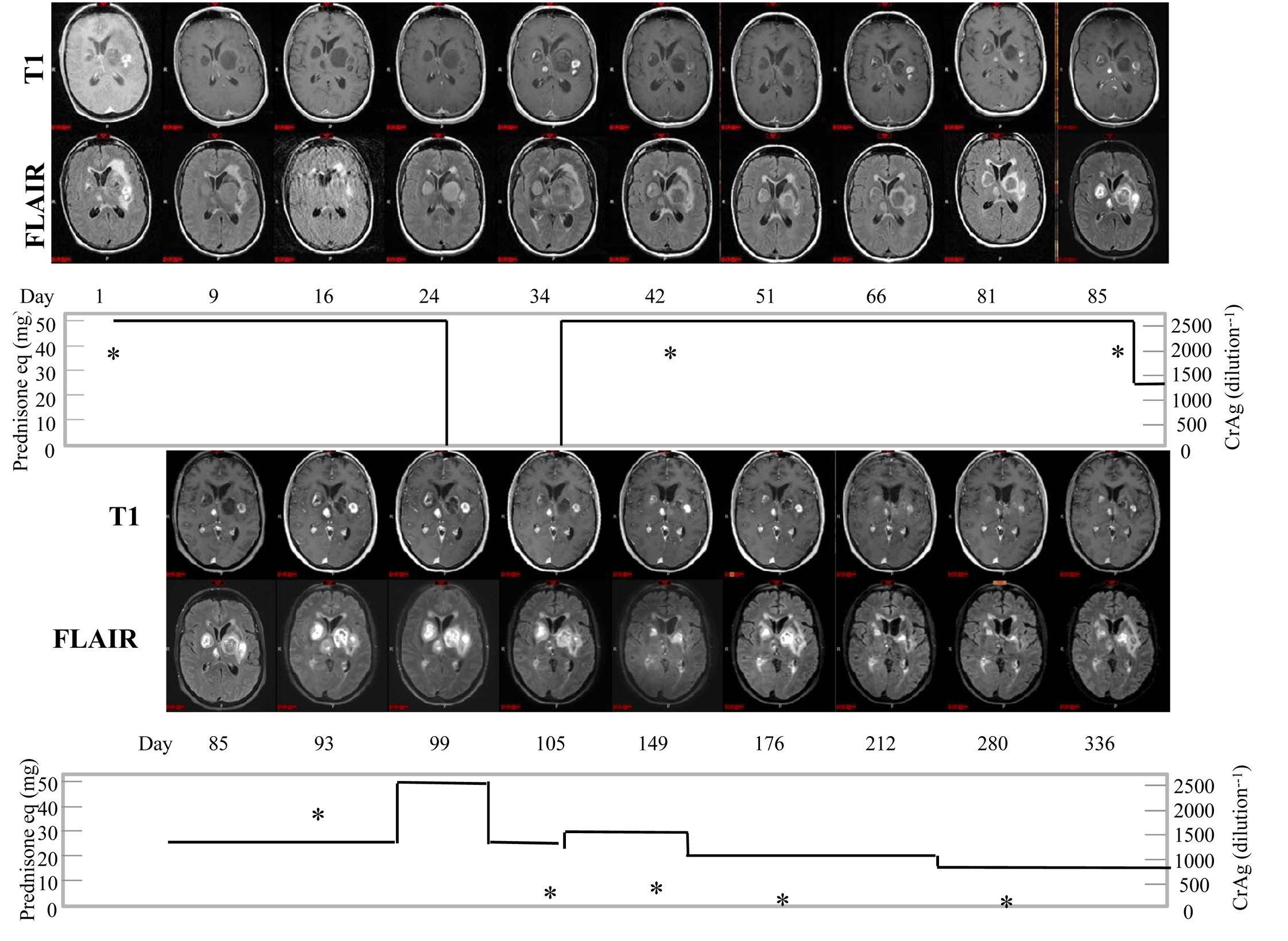

Supplement: S1 Fig — Top panel, Enhanced MRI T1 and FLAIR weighted images corresponding to hospital Day. Lower panels, indicated steroid dosage (black line) and CSF cryptococcal latex antigen titer (*). (TIF) [file ppat.1004884.s001.tif]
