# Supplementary material for: Paradoxical Immune Responses in Non-HIV Cryptococcal Meningitis
Source: PLoS Pathog. 2015 May 28;11(5):e1004884. doi: 10.1371/journal.ppat.1004884 (PMC4447450; doi:10.1371/journal.ppat.1004884)
Supplement: S1 Table — (DOCX) [file ppat.1004884.s004.docx]

|  | | | | |
| --- | --- | --- | --- | --- |
|  | Conjugation | Name | Company (Clone) |  |
|  | FITC | Anti-human CD56 antibody | BD (Clone: MEM188) |  |
|  | PE | Anti-human SCF R/ c-kit antibody | R&D (Clone: 47233) |  |
|  | PerCP-Cy5.5 | Anti-human CD123 antibody | eBioscience (Clone: 7G3) |  |
|  | PE-Cy7 | Anti-human CD11c antibody | eBioscience (Clone: 3.9) |  |
|  | V450 | Anti-human CD45 antibody | BD (Clone: HI30) |  |
|  | AmCyan | Anti-human CD8 antibody | BD (Clone: SK1) |  |
|  | eFluor 605 Nanocrystal | Anti-human CD19 antibody | eBioscience (Clone: HIB19) |  |
|  | eFluor 655 Nanocrystal | Anti-human CD3 antibody | eBioscience (Clone: OKT3) |  |
|  | Qdot 705 | Anti-human CD4 antibody | Invitrogen (Clone: S3.5) |  |
|  | APC | Anti-human TCRγδ antibody | BD (Clone: B1) |  |
|  | Alexa Fluor 700 | Anti-human CD14 antibody | BioLegend (Clone: HCD14) |  |
|  | APC-Cy7 | Anti-human HLA-DR antibody | eBioscience (Clone: LN3) |  |
|  |  |  |  |  |
